# Visual outcomes of primary keratoprosthesis implantation in transplant-naïve eyes

**DOI:** 10.1371/journal.pone.0311413

**Published:** 2024-10-03

**Authors:** Camryn Thompson, Cason Robbins, Rami Gabriel, C. Ellis Wisely, Melissa Daluvoy, Sharon Fekrat

**Affiliations:** Department of Ophthalmology, Duke University School of Medicine, Durham, North Carolina, United States of America; Harvard Medical School, UNITED STATES OF AMERICA

## Abstract

**Purpose:**

Primary keratoprosthesis (Kpro) implantation may be indicated in eyes that have an expected poor prognosis following initial penetrating keratoplasty, such as eyes with limbal stem cell deficiency (LSCD). We compare visual outcomes of eyes undergoing primary Kpro to eyes that had a secondary Kpro following penetrating keratoplasty.

**Methods:**

Retrospective review of all patients who had Kpro implantation at a tertiary academic medical center from 2005–2020. Among those, eyes that had undergone primary Kpro implantation without a history of prior corneal transplantation were also identified.

**Results:**

Eighty-four eyes of 77 patients that had undergone Kpro implantation were identified. Of those 84, 12 eyes (21.4%) of 12 patients were receiving primary Kpro since they were corneal transplant-naïve. Among individuals undergoing primary Kpro implantation compared to secondary Kpro implantation, the most common underlying diagnoses were limbal stem cell deficiency (41.7% vs 10.0%, p = 0.01304), corneal scarring not otherwise specified (25.0% vs 2.86%, p = 0.02077), and neurotrophic cornea (16.7% vs 2.86%, p = 0.1002). Eyes undergoing primary Kpro implantation had similar mean visual acuity to eyes undergoing secondary Kpro preoperatively (20/2118 vs 20/3786, p = 0.271), 3 months postoperatively (20/264 vs 20/758, p = 0.174), and at final follow up (average 3.06 years, 20/907 vs 20/3446, p = 0.070). Average follow-up time and rates of glaucoma, endophthalmitis, retroprosthetic membrane, and retinal detachment did not significantly differ between groups (all p > 0.05). All eyes that progressed to no light perception (n = 13) had undergone secondary Kpro implantation.

**Conclusions:**

Visual acuity outcomes were similar between primary Kpro implantation and secondary Kpro implantation. Eyes that underwent primary Kpro implantation trended toward better postoperative VA at final follow-up than secondary Kpro eyes.

## Introduction

Penetrating keratoplasty (PK) can significantly improve visual acuity in patients with visually significant corneal pathology including stromal corneal opacity and corneal ectasia [1]. While PK involves transplantation of a full-thickness corneal graft from a donor, an alternative surgical option is placement of a permanent keratoprosthesis (Kpro), an artificial corneal implant usually reserved for eyes with severe corneal disease and an otherwise poor visual prognosis following PK [2]. Indications for Kpro implantation have traditionally included herpes keratitis with associated scarring and neovascularization, chemical burns, autoimmune diseases and repeat PK failures [3–8].

Despite proven efficacy, Kpro implantation is still often considered a surgical ‘last resort’ for eyes with extensive anterior segment pathology after failed PK [9]. This approach is mostly a result of the potential risks associated with Kpro in both the immediate and longer-term postoperative period, including retroprosthetic membrane (RPM) formation, endophthalmitis, keratitis, and glaucoma [8, 10–14]. Currently, one of the most common indications for Kpro placement is prior PK failure [11, 15, 16]. More recent studies, however, have demonstrated longer-term visual improvement in eyes following both primary and secondary Kpro implantation [17, 18].

Some studies have explored the use of primary Kpro implantation as the initial intervention of choice for some eyes with severe corneal pathology [15, 19–23]. Studies have demonstrated that primary Kpro implantation can result in rapid and sustained visual improvement with a similar risk profile to that in high-risk eyes undergoing PK [21, 24, 25]. Comparison of eyes undergoing primary and secondary Kpro demonstrated that eyes with primary Kpro are more likely to have sustained improvement in visual acuity compared to eyes with secondary Kpro implantation [20–22]. Additionally, eyes with preexisting ophthalmic conditions including corneal neovascularization, herpetic keratitis, and limbal stem cell deficiency (LSCD) in which PK has been associated with poor outcomes, primary Kpro was associated with a significantly greater percentage of eyes with visual acuity ≥ 20/200 in the first 4 years [23].

In this study, we report a diverse cohort of eyes that underwent primary Kpro implantation and assess preoperative indications, postoperative complication rates, and long-term visual outcomes.

## Methods

This cross-sectional study was reviewed and approved by the Duke University Health System Institutional Review Board (IRB) (Pro00105461). The requirement for written informed consent was waived due to the retrospective nature of the study. This study adhered to the tenets of the Declaration of Helsinki regarding enrollment of human subjects as well as information privacy as defined in the Health Insurance Portability and Accountability Act of 1996 (HIPAA). Individual participants could be identified during data collection, then all patient data was de-identified for subsequent storage and analysis. Protected health information was stored in a secure study folder behind a Duke firewall or within DukeBox, which was accessible online to secure Duke accounts of IRB-approved personnel. Clinical data was accessed between 01/01/2021 and 12/31/2023.

Retrospective chart review was conducted using the Duke Enterprise Data Unified Content Explorer (DEDUCE) to identify all individuals with a history of Kpro implantation (CPT code 65770) [26]. DEDUCE was accessed within a secure workspace called Protected Analytics Computing Environment (PACE) through Duke University Health System. Medical records were manually reviewed to exclude individuals who had a temporary keratoprosthesis used intraoperatively. All individuals with a history of Boston Kpro type 1 implantation were included for further analysis. Collected clinical data included preoperative ocular comorbidities, prior ocular surgeries, follow-up duration, postoperative complications, and both preoperative and postoperative corrected visual acuity. Age and sex were recorded as documented in the electronic medical record at the time of the relevant surgery. Individuals with no prior history of corneal transplantation were identified for the purposes of this study to compare outcomes between those undergoing primary Kpro implantation and those undergoing secondary Kpro implantation following prior PK. A Kpro is considered primary if it is the first ever corneal transplant in an eye, and a secondary Kpro is any Kpro implantation occurring after a prior corneal transplant or multiple prior corneal transplants. Snellen equivalent visual acuity was converted to the logarithm of the minimum angle of resolution (logMAR) for the purposes of statistical comparison. The logMAR equivalents of counting fingers, hand motion, light perception, and no light perception (NLP) were estimated to be 2.3, 2.6, 2.9, and 3.2, respectively, as extrapolated from previous work that estimated that each stage of “off-the-chart” visual acuity represented a doubling of the visual angle [27]. The logMAR visual acuity was then converted back to Snellen equivalent for ease of clinical comparison.

All analyses were conducted using Stata 14.0 (StataCorp LLC, College Station, TX, USA) and RStudio (Posit PBC, Boston, MA, USA). Categorical variables were compared between groups utilizing the Fisher’s Exact Test and means were compared with two-tailed t-tests. The value of statistical significance was set at p < 0.05.

## Results

A total of 84 eyes of 77 patients were identified as having undergone permanent Boston Kpro implantation between 2005 and 2020. Of these 84 eyes, 12 eyes (21.4%) of 12 patients underwent Boston Kpro implantation as a primary procedure without prior corneal transplantation. Among patients undergoing primary transplant, average age at time of surgery was 68.9 years (± 15.5 years), and 41.7% of patients were female, and 58.3% of patients were male. Among patients undergoing secondary transplants, the average age at time of surgery was 62.7 years (± 17.1 years), and 40.5% of patients were female and 59.5% were male. Eyes classified as secondary Kpro had a mean and mode of 3 prior transplants, and the minimum, median, and maximum number of transplants were 1, 2, and 8, respectively (S1 Table). Neither age nor sex were significantly different between the two groups (p = 0.244 and p = 0.242 respectively).

Among individuals undergoing primary Kpro implantation, the most common underlying diagnoses for Kpro placement limbal stem cell deficiency (LSCD), including 2 aniridia cases (41.7% vs 10.0%, p = 0.01304), corneal scarring not otherwise specified (25.0% vs 2.86%, p = 0.02077), and neurotrophic cornea (16.6% vs 2.86%, p = 0.1002) (Table 1). The most common underlying diagnoses for secondary transplant were LSCD as above, including 5 aniridia cases; chemical burn (12.9% vs 8.33% for primary, p > 0.99); pseudophakic bullous keratopathy (10.0% vs 8.33% for primary, p > 0.99); and trauma (11.4% vs 0.0% for primary, p = 0.5852) (Table 1).

**Table 1:** Underlying diagnoses for cornea transplant.

	Primary KPro n (%) (n = 12 eyes)	Secondary KPro n (%) (n = 70 eyes)	Fisher’s Exact Test p-value
**Limbal stem cell deficiency**	5 (41.7%)	7 (10.0%)	**0.01304**
**Neurotrophic cornea**	2 (16.6%)	2 (2.86%)	0.1002
**Chemical burn**	1 (8.33%)	9 (12.9%)	>.99
**Traumatic injury**	0 (0%)	6 (11.4%)	.5852
**Fuchs’ dystrophy**	0 (0%)	4 (5.71%)	>.99
**Stevens-Johnson syndrome**	0 (0%)	3 (4.29%)	>.99
**Pseudophakic bullous keratopathy**	1 (8.33%)	7 (10.0%)	>.99
**Corneal scar, unspecified**	3 (25.0%)	2 (2.86%)	**0.02077**

Eyes undergoing primary Kpro implantation had similar mean visual acuity to eyes undergoing secondary Kpro preoperatively (20/2118 vs 20/3786, p = 0.271), 3 months postoperatively (20/264 vs 20/758, p = 0.174), and at final follow up (average 3.06 years, 20/907 vs 20/3446, p = 0.070) (Table 2).

**Table 2:** Visual outcomes of primary and secondary KPro placement.

	Primary KPro logMAR (Snellen)	Secondary KPro logMAR (Snellen)	T-test P-value
**Preoperative visual acuity (VA)**	2.025 (20/2118)	2.277 (20/3786)	0.27080
	*n = 12*	*n = 71*	
**VA at 3 Months**	1.120 (20/264)	1.579 (20/758)	0.17353
	*n = 9*	*n = 63*	
**VA at 12 Months**	1.575 (20/752)	1.571 (20/745)	0.99251
	*n = 8*	*n = 60*	
**VA at Final Follow-Up** (average 3.06 years)	1.657 (20/907)	2.221 (20/3446)	0.06998
	*n = 12*	*n = 65*	

Average follow-up time of 3.06 years and rates of postoperative complications including glaucoma, endophthalmitis, RPM formation, and retinal detachment did not significantly differ between the 2 cohorts (all p > 0.05). However, all eyes that progressed to NLP vision or enucleation (n = 13, n = 6 respectively) had undergone secondary Kpro implantation (Table 3).

**Table 3:** Complications of Kpro implantation.

Group	Endophthalmitis n (%)	Retroprosthetic Membrane n (%)	YAG to Treat RPM (n instances)	PPV to Treat RPM n (%)	Recurrence of RPM after YAG n (%)	Sterile Vitritis n (%)	Retinal Detachment n (%)	Retina Surgery after Kpro Surgery n (%)	Final visual acuity NLP n (%)	Enucleation n (%)
**Primary Kpro**	0 (0%)	4 (33.3%)	1	1 (8.33%)	0 (0%)	0 (0%)	2 (16.7%)	2 (16.7%)	0 (0%)	0 (0%)
**Secondary Kpro**	6 (8.57%)	30 (42.9%)	30	15 (21.4%)	9 (12.9%)	1 (1.43%)	10 (14.3%)	13 (18.6%)	13 (20.0%)	7 (9.72%)
**P-Value** *	0.5852	0.7527	0.252	0.4442	0.3435	>.99	>.99	>.99	0.2011	0.5861

*P-values for all variables except for YAG to treat RPM are based on Fisher’s Exact Test. P-value for YAG to treat RPM is based on a two-tailed T-test.

YAG: Yttrium Aluminum Garnett, PPV: Pars plana vitrectomy, NLP: No light perception

In the primary KPro group, the 2 eyes with aniridia developed retroprosthetic membranes. In the secondary KPro group, 3 of the 5 eyes with aniridia developed retroprosthetic membranes. Four of the 12 eyes that underwent primary Kpro progressed to LP vision at final follow-up, and of those eyes, primary indications were 1 neurotrophic ulcer, 2 LSCD, 1 unspecified corneal scar.

## Discussion

In this study, we demonstrate a trend towards better visual acuity at final follow-up in patients undergoing primary Kpro compared with patients undergoing secondary Kpro implantation, despite similar preoperative visual acuity across cohorts. Postoperative complications were also not significantly different across groups, but there was a trend of differences in RPM management, potentially related to more RPM recurrences in the secondary Kpro cohort and physician preference. There were significantly different preoperative diagnoses across cohorts; most notably, many patients in the primary Kpro group had LSCD, which included 2 cases of aniridia. Eyes with LSCD are likely favored to undergo Kpro upfront, as this approach negates the need for limbal stem cell transplantation alongside a traditional corneal transplant [28]. Without the associated limbal stem cell transplant, eyes with LSCD have reduced ability to repair and heal the traditional grafts because, by definition, eyes with LSCD lack adequate stem cell proliferative potential. Thus, this study also concludes that patients with severe LSCD may be able to avoid an additional procedure in favor of primary Kpro, which yields similar visual outcomes and complication rates as secondary Kpro.

Our findings are consistent with a study by Fadous and colleagues who reported on 30 patients undergoing primary Kpro and 40 patients undergoing secondary Kpro with a single surgeon and noted that the primary Kpro group had significantly better visual acuity at one year [21]. Follow-up time was one year in the study by Fadous and colleagues, while this study had an average follow-up time of over three years, providing more longitudinal insights [21]. For example, this study captured later outcomes not described by Fadous and colleagues, such as enucleation in some secondary Kpro eyes. Our study also includes worse preoperative visual acuity in both primary and secondary Kpro cohorts compared to the eyes in the study by Fadous by several lines, representing a slightly different patient population. While Fadous and colleagues had more balanced cohort sizes, there were significant differences in age between their cohorts, which was not the case in our study.

In another study by Driver and colleagues, a total of 262 Kpros implanted by two surgeons were analyzed [23]. This study corroborated that visual outcomes tended to be better in the primary Kpro group, with more patients having better than 20/200 vision in the primary group at 1 year (84.8% vs 67.4%, p = 0.01). This finding is similar to our study, which demonstrated that patients with primary Kpro implantation had a trend of better visual acuity at final follow-up. The study by Driver and colleagues has a longer follow-up time of 6 years; however, their study does not present the shorter-term visual outcomes presented in our study or the underlying diagnoses associated with worse visual outcomes in the primary Kpro cohort, which may be an important visual prognostic factor [28]. Underlying diagnoses also have an impact on complications; for example, initial diagnoses of infectious keratitis and aniridia are associated with higher rates of retroprosthetic membranes [10]. Significant representation of these diseases in patient cohorts can therefore impact outcomes comparisons. Moreover, the ages between their cohorts are significantly different (mean ± SD: 47.1 ± 18.1 primary vs. 61.2 ± 19.1 years old secondary, *P* < 0.0001) [23], which presents a potential confounder that is not present in our study.

This study is limited by its retrospective nature as well as its lack of a comparison group of individuals undergoing PK without Kpro. Additionally, significantly more patients in our study underwent secondary Kpro implantation compared to primary Kpro implantation, and our primary Kpro group was relatively small, hindering further sub-analyses. Finally, our study included Kpro implantation from more than one surgeon which may introduce uncontrollable variance into our findings. However, strengths of this study include similar baseline characteristics between cohorts, including age and preoperative visual acuity. Our analysis also provides both short- and intermediate-term visual outcomes, which was not the case in other studies [21, 23, 29]. Another unique aspect of our study is worse preoperative visual acuity in both cohorts compared to previous studies, representing a patient population with more visually significant pathology before surgery [21–23].

The data presented herein is a valuable contribution to the scarce body of published literature on the use of primary Kpro for visual rehabilitation. Only about a thousand Kpros are implanted worldwide each year, whereas there are over 200,000 penetrating keratoplasties performed yearly [30, 31]. The Kpro appears to be less commonly utilized, likely due in part to it being reserved as a ‘last resort” therapeutic option, surgeon unfamiliarity with implantation technique, and potential risk for complications. There are only a handful of studies on the use of Kpros as a first-line intervention for severe corneal disease [30], and thus additional informative publications on primary Kpro may promote further interest in exploring its use and instill more confidence in the adoption of this technique.

Overall, this study corroborates findings of prior studies indicating that primary Kpro implantation may have long-term visual benefits with a similar complication profile to secondary Kpro, while contributing both short- and long-term visual outcomes in a single patient population, as well as comparisons between primary and secondary Kpro cohorts with similar baseline characteristics. Given the known complications of Kpro implantation compared to PK, including RPM formation and elevated intraocular pressure with glaucomatous progression, patients undergoing primary Kpro must be carefully selected and closely monitored postoperatively. Further advancements in the design of the Kpro may lead to improved outcomes. A prospective study examining the benefits of primary Kpro implantation versus PK, particularly in high-risk groups, may yield further insight.

## Supporting information

S1 Table De-identified patient characteristics and outcomes. (XLSX)
